# 3D Cell Printing of Tissue/Organ-Mimicking Constructs for Therapeutic and Drug Testing Applications

**DOI:** 10.3390/ijms21207757

**Published:** 2020-10-20

**Authors:** Jongmin Kim, Jeong Sik Kong, Wonil Han, Byoung Soo Kim, Dong-Woo Cho

**Affiliations:** 1 Department of Mechanical Engineering, Pohang University of Science and Technology (POSTECH), Pohang 37673, Korea; mandarinbear@postech.ac.kr; 2 School of Interdisciplinary Bioscience and Bioengineering, Pohang University of Science and Technology (POSTECH), Pohang 37673, Korea; urhere@postech.ac.kr; 3 Division of Integrative Biosciences and Biotechnology, Pohang University of Science and Technology (POSTECH), Pohang 37673, Korea; wihan@postech.ac.kr; 4 Future IT Innovation Laboratory, Pohang University of Science and Technology (POSTECH), Pohang 37673, Korea; poohtw77@postech.ac.kr; 5 Institute of Convergence Science, Yonsei University, Seoul 03722, Korea

**Keywords:** 3D cell printing, bioink, tisse engineering, regeneration medicine, drug testing platform

## Abstract

The development of artificial tissue/organs with the functional maturity of their native equivalents is one of the long-awaited panaceas for the medical and pharmaceutical industries. Advanced 3D cell-printing technology and various functional bioinks are promising technologies in the field of tissue engineering that have enabled the fabrication of complex 3D living tissue/organs. Various requirements for these tissues, including a complex and large-volume structure, tissue-specific microenvironments, and functional vasculatures, have been addressed to develop engineered tissue/organs with native relevance. Functional tissue/organ constructs have been developed that satisfy such criteria and may facilitate both in vivo replenishment of damaged tissue and the development of reliable in vitro testing platforms for drug development. This review describes key developments in technologies and materials for engineering 3D cell-printed constructs for therapeutic and drug testing applications.

## 1. Introduction

Since the first cell culture was reported by Ross Harrison, cellular engineering has advanced through the development of various animal/plant cell culture methods, the establishment of useful cell lines, the discovery of stem cells, and reprogramming technologies for the production of induced pluripotent stem cells (iPSCs) and other cells of interest [1,2]. When 3D cultivation methods were reported in the early twenty-first century, various biomaterials and scaffolds were introduced to the conventional cell culture techniques to provide 3D environments beyond the culture plate and modulate behaviors of the cells [3,4,5]. The cell-based constructs using these complex approaches have shown improved performance compared to conventional 2D culture methods by providing an environment that mimics native tissue. Many studies have also been conducted on the feasibility of using this technique to develop functional tissue/organs in the field of tissue regeneration and in vitro models [6,7,8].

An early type of cell-based construct was fabricated by seeding cells on a porous scaffold to be used for clinical and pharmaceutical applications [9,10,11]. Various techniques and materials were used to fabricate the tissue/organ mimetic structure, the scaffold, and then cells were seeded manually [12,13,14]. Using this method, several types of tissue, including cartilage, bone, and skin were developed and showed significant advances in the treatment of partial defects [15,16,17]. However, due to limitations in the areas of multi-cellular interaction, vascularization, and the creation of a complex physiological environment, there are challenges in regenerating large defects and developing therapeutics for native tissue/organs with complex cell arrangements and architectures such as the liver, lung, and kidney.

To overcome these limitations, 3D cell printing has been researched due to its ability to precisely deposit various biomaterials and living cells according to their native equivalents [18,19,20]. The 3D structure is fabricated by stacking 2D patterns in layers during the printing process. This automated system provides a precisely controlled structure with high reproducibility [21,22,23]. Moreover, advanced control systems have enabled 3D printing techniques to dispense live cells [24,25]. Advances in medical imaging technology, including computed tomography and magnetic resonance imaging, have also improved 3D cell printing [26,27,28]. These new technologies facilitate the fabrication of complex structures via computer-aided design (CAD) and computer-aided manufacturing (CAM) processes based on medical imaging.

In addition to novel fabrication methods, the development and advancement of bioink, which is a printable biomaterial capable of encapsulating cells, has also contributed to preserving 3D structure, providing a suitable biochemical environment for printed cells, and protecting cells from harsh conditions during the printing process [29,30]. Therefore, the combination of 3D cell printing technology and functional bioinks could meet the physical, chemical, and biological requirements for developing living tissue/organs [31]. To date, various types of 3D-printed functional tissue/organs have shown the potential for successful transplantation [32]. Also, the printed construct has been studied for in vitro tissue/organ models for testing platforms in the pharmaceutical industry to detect drug-induced toxicity or to validate the drug efficacy for the target diseases [33]. These applications of 3D printing techniques are expected to advance drug development and reduce the number of patients suffering from a global organ shortage.

This review introduces the essentials of printing 3D constructs through selected printing methods and bioinks for therapeutic application and drug testing platforms. The mechanisms of various 3D cell printing techniques and advanced bioinks are described, followed by their advantages and disadvantages. The specific applications of 3D printed tissue/organs in the areas of therapeutics and drug testing platforms are also presented. Finally, the review discusses the challenges and future areas of study for 3D cell printing technology and bioinks.

## 2. Printing Methods

Since it was first described by Charles W. Hull as stereolithography (SLA), 3D printing has been widely studied as a new manufacturing system and showed greater versatility compared to conventional processes such as molding and milling [34]. This technique has been used in the development of biomedical tools and the fabrication of microfluidics, or organ-on-a-chip [35]. There are several types of printing methods depending on the material used, which may be a metal, polymer, or biomolecule [36]. However, not all of these methods can be used in 3D cell printing, since the cells are encapsulated in a hydrogel, called bioink, to prevent shear damage during the printing process. This review describes the methods and materials used in 3D cell printing for tissue engineering and other applications (Table 1).

### 2.1. Laser Induced Forward Transfer-Based Printing System

The laser-induced forward transfer (LIFT) system is composed of a pulsed laser source, a laser-absorbing layer with a cell-containing hydrogel layer, and the receiving substrate [44,45]. The laser source usually has an ultraviolet wavelength with a nanosecond pulse. Glass or quartz is used in the laser-absorbing layer since these materials are transparent for laser irradiation. The bioink layer or cell culture medium covers the receiving substrate to hold the printed cells. With the use of CAD/CAM, the pulse of the laser can be focused on a small area of the laser-absorbing layer and generate a vapor pocket using high pressure. Then the cell-laden droplet forms, falls down to a receiving substrate, and crosslinks. Although this technique requires a complex system, including pulse control of the laser to determine the thickness of each layer and determining the distance between the hydrogel layer and the substrate, the LIFT-based system has high resolution and reproducibility. These qualities make this system suitable for printing a single cell or cell arrays [24,37]. It can also be used to stack different cell layers to create a multi-cellular construct.

Because of these characteristics, this system has been used in various biomedical applications [46]. For example, a skin model composed of fibroblasts and keratinocytes was developed [47]. Each cell was encapsulated in collagen bioink and dispersed onto a sheet of Matriderm. The printed model showed a compartmentalized structure and intercellular communication over the gap junction. Ovsianikov et al. developed a multicellular structure with endothelial and vascular smooth muscle-like cells [48]. Each cell was encapsulated in plasma/alginate bioink and dispersed onto the poly(ethylene glycol) diacrylate scaffold. The structure showed a sharp transition region between the endothelial and smooth muscle area. Martin et al. printed adipose-derived stem cells (ASC) and endothelial colony-forming cells (ECFC) for the 3D array. Intercellular communication was observed due to the formation of a vascular network between ASC and ECFC. Additionally, since the system is a non-contact or nozzle-free process, it avoids clogging and enables the use of relatively higher concentrations of bioink and cells [49]. However, risk factors include damage to the cell from direct exposure to the laser and absorbed energy in the substrate [20,38,50].

### 2.2. SLA-Based Printing

**Figure 1:**
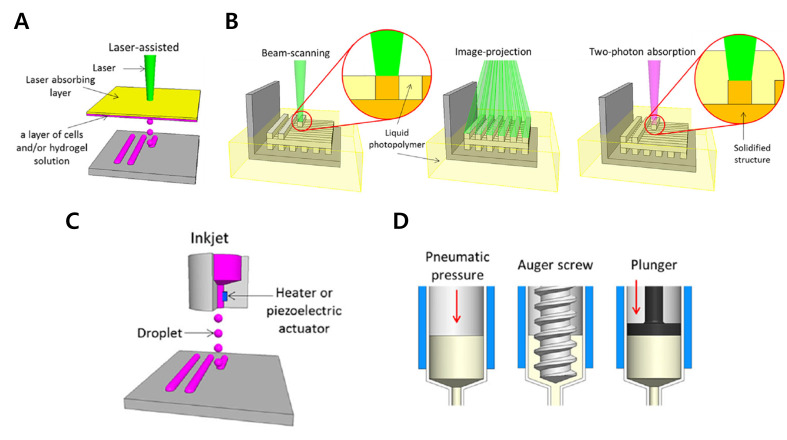
Schematic image of printing methods. (**A**) L*IFT*-based printing system. (**B**) SLA-based printing system. (**C**) Inkjet-based printing system. (**D**) Extrusion-based printing system (reproduced with permission from ref [20]; copyright 2016 Springer Nature).

SLA was the first reported printing technique that could fabricate complex 3D structures with high resolution and accuracy [41]. Similar to the LIFT-based printing system, SLA uses a light source to crosslink or polymerize the bioink to form the 3D structure (Figure 1B). It writes the 2D pattern directly by selective polymerization of photocurable bioinks through light exposure with a wavelength ranging from ultraviolet to visible light. The 3D structure is obtained by stacking 2D patterns through a layering process [39]. The resolution depends on the spot size of the laser and the absorption range of the wavelength of the bioink [51]. There are two approaches for polymerization: beam-scanning and image projection [52,53]. The beam-scanning technique simply “draws” the pattern with a laser, and the polymerization of bioink is initiated along the laser path. The image projection method uses a 2D image, generated by a digital micromirror, to polymerize the entire pattern with a single projected image, which reduces the printing time. The optimized light system and optics devices used with SLA facilitate the printing of microscale 3D structures [54,55,56].

Xuanyi et al. used the SLA method to print a 3D hexagonal liver lobule-like structure with iPSC-derived hepatic cells, human umbilical vein endothelial cells (HUVECs), and adipose-derived stem cells [57]. The hepatocytes cultured in the patterned model showed an increase in liver-specific markers and the secretion of metabolic products. In another study, Soman et al. developed a 3D cell-laden microstructure with GelMA and fibroblasts/mesenchymal progenitor cells [58]. Various shapes of the cell-laden microstructure were printed, including spiral, pyramid, flower, and dome shapes. The encapsulated cells proliferated well and expressed their marker in all geometries.

With the adoption of two-photon polymerization (2PP), SLA can achieve 100 nm resolution [59,60,61,62]. This resolution can be obtained by absorbing two photons at the same time and point. The two absorbed photons act as a single photon but with double the wavelength, and this phenomenon induces polymerization in a small region without disturbing other areas. The high resolution makes the 2PP technique suitable for biomedical applications [63,64,65,66]. Kufelt et al. developed a methacrylate-modified hyaluronic acid (HAGM)-based scaffold for bone tissue engineering applications [67]. The fabricated scaffold showed high swelling ability and biocompatibility. Additionally, the HAGM was functionalized with epidermal growth factor to enhance the proliferation of fibroblasts. Due to these characteristics, SLA printing has been used in tissue engineering and regenerative medicine [68,69,70]. The major drawback of this system is that it risks cellular damage from the photocurable bioink and ultraviolet laser [71].

### 2.3. Inkjet-Based Printing

Inkjet printing is a contactless method that ejects a small droplet of bioink and cell from the inkjet-head nozzle onto the substrate (Figure 1C). There are two types of droplet generation: thermal and piezoelectric [72]. The thermal inkjet head contains a micro heater, and it vaporizes the bioink with a temperature range of 100~200 °C to form a droplet [73]. The piezoelectric type ejects the bioink by mechanical force from the piezoelectric actuator to form a droplet at regular intervals [74]. Since both types are commonly used in commercialized inkjet printers, mass-produced inkjet heads, such as Hewlett-Packard and Epson products, have been modified by replacing their ink with bioink containing cells [75,76,77,78]. This system offers high resolution (20~60 μm), a small droplet volume (1~100 pl), and high speed (1~10,000 drops/s), enabling a high-throughput printing system [79,80,81,82]. However, this printing system limited to low-viscosity bioink and is susceptible to nozzle clogging and unstable printing at a high-throughput rate [83].

### 2.4. Extrusion-Based Printing

Extrusion printing is currently the most common system used in both biological and non-biological applications. It originated from fused deposition modeling (FDM), which extrudes molten polymer using pneumatic or piston-derived forces and stacks layer-by-layer to fabricate 3D structures (Figure 1D). The pneumatic system extrudes material using compressed air, and the piston system uses mechanical movement. Both systems can dispense more viscous material such as polycaprolactone (PCL) and poly (lactic-co-glycolic) acid (PLGA). Using this thermoplastic polymer, the extrusion printing method has been widely used to fabricate scaffolds and develop drug-releasing platforms [84,85,86].

Advancements in the precise control of movement and dispensing force have enabled the extrusion printing system to print low-viscosity materials at the microscale [87,88,89]. This advanced controlling system facilitates the printing of cells in a 3D structure similar to their natural arrangement. Compared to other methods, the extrusion method is a slower printing process. The LIFT-based and SLA methods can change the laser focus quickly, enabling high printing speeds, and the inject method ejects 10,000 droplets per second. However, these methods only work with photocurable or low viscosity bioink, which can damage the cells and limit the height of the printed layer, respectively. The extrusion method can be used with a wide range of printing materials, including thermoplastic polymers and hydrogels, and their printing characteristics can be easily modified by changing the size of the nozzle and regulating pressure/movement at the extrusion chamber.

Moreover, a multi-head system can print various types of materials simultaneously [90,91]. Since each material has distinct properties, the optimal printing condition can be adjusted for each material, expanding the ability to print a wide range of materials for biomimetic tissue constructs [92,93]. For example, one study used a thermoplastic polymer to print a scaffold or chip platform, and then cells in bioink were printed for regenerative medicine or organ-on-a-chip applications [94,95,96].

Advanced printing techniques based on the extrusion method also have been reported. Hydrogel-based bioinks have been used for cell printing because they support viability, proliferation, and differentiation. However, due to their low viscosity, a structure with a high aspect ratio is difficult to achieve. The freeform reversible embedding of suspended hydrogels (FRESH) method has been proposed to obtain a stable 3D structure with a hydrogel-based bioink [97,98]. In the FRESH method, the bioink was is printed into a support bath composed of a gelatin microparticle slurry. This supporting bath acts like a Binhgham plastic during the printing process to maintain the printed structure during its complete gelation. Lee et al. used this technique to printed a human cardiac ventricle model using collagen and human embryonic stem cell-derived cardiomyocytes and cardiac fibroblasts [99]. This millimeter-scale ventricle model maintained its structure for up to 28 days and showed synchronized contractions and directional action potential propagation. In addition, a neonatal-scale human heart was printed, and patient-specific anatomical structure was determined by micro-CT imaging. In another example of printing using a gelatin-based support bath, the 3D cell-printed skeletal muscle construct was successfully printed while maintaining its organized volumetric structure that was mainly composed of muscle-derived tissue-specific bioink [100]. In vitro and in vivo results for the model showed high viability, aligned muscle fiber structure, and restoration of volumetric muscle loss from an injured tibialis anterior.

In other studies, a coaxial nozzle was used to print a fiber structure with multiple layers [101,102]. The thickness of each layer can be adjusted by combining nozzles of different diameters or controlling the pressure of each chamber [103,104]. One of the major advantages of coaxial printing is the ability to fabricate a hollow structure using sacrificial material in the core part. Using this technique, an artificial blood vessel was printed directly and implanted into an animal model [105].

The extrusion-based printing system is also versatile because it can be combined with other methods such as inkjet-based printing. Since both systems have a similar structure with a different type of printing head, they can be combined into an integrated system that can fabricate both a large volume of cellular structure and a monolayer of cells. For example, a combination of the multi-head extrusion system and the inkjet-system was used to fabricate an in vitro skin model composed of cellular layers of different thicknesses [106]. Photopolymerization devices can also be integrated with this system. The use of a UV illuminator and a photocurable bioink, such as gelatin methacryloyl or polyethylene glycol diacrylate, can facilitate the crosslinking of printed bioink prior to ejection to form a stable structure [107,108].

## 3. Bioink

Depending on the printing method, bioink has to meet the requirements for viscosity, mechanical strength, and polymerization properties to enable 3D lamination by the printing process while protecting cells from stress and external risk factors during the printing process. In addition, bioinks are a key variable in supporting cell adhesion, growth, and development. Accordingly, it is important to select a bioink that is suitable for the printing method and the target tissue being produced. The following table describes various types of bioinks and their suitability for different printing methods and crosslinking methods, cell viability, and their advantages and disadvantages (Table 2).

### 3.1. Synthetic Polymer-Based Bioinks

When manufacturing bioink that contains a synthetic polymer, it is easy to control the mechanical properties, crosslinking method, and biocompatibility. The main synthetic polymers used for 3D cell printing include poly(ethylene glycol) diacrylate (PEGDA), poly(ethylene glycol)-tetraacrylate (PEGTA), and poly(caprolactone) (PCL). PEGDA and PEGTA are mainly used for SLA-based printing. PEGTA generally has better rheological properties and cellular activity than PEGDA, and these properties can be controlled by altering the molecular weight of PEG [110]. PCL has mainly been used as a structural supporting material in extrusion-based bioprinting because it has high mechanical properties, a low glass conversion temperature of about 60 °C, and rheological properties suitable for extrusion printing. However, due to the problem of the material source, synthetic polymer-based bioinks cannot recapitulate the actual ECM environment, so their ability to promote cell adhesion, survival, and maturation is poor [125]. In addition, the cytotoxicity of synthetic biomaterials impairs cell viability and function, limiting the long-term use of in vitro models. These weaknesses limit their applications in 3D cell printing.

### 3.2. Carbohydrate Polymer-Based Bioinks

Alginate, agarose, and Gellan gum are used as carbohydrate polymer-based bioinks. They are made by processing non-mammalian organisms, are inexpensive, and have massive productivity. In the case of alginate, a divalent cation such as calcium is used as the crosslinking agent. A rapid crosslinking reaction occurs when calcium ions are diffused into an alginate solution, making it a widely-used material in 3D cell printing [30]. Agarose is a thermosensitive hydrogel made by seaweed extraction. It has a low melting point, maintaining a liquid state at a temperature of 60 °C or higher and has a gelling property at a temperature of 30 °C or lower. Gellan gum has the gelling property when combined with a divalent cation such as calcium and magnesium at physiological temperature. Bioinks based on carbohydrate polymers are helpful for simplifying the printing process because of the simple crosslinking method using heat or a divalent cation. However, since there is no binding site for transmembrane cell proteins, carbohydrate-based bioinks have lower cell adhesion, survival, and growth. To overcome this limitation, researchers carried out a study to immobilize the tripeptiede Arg-Gly-Asp (RGD) motif to the polysaccharide chain of the carbohydrate polymer. The RGD motif acts as a ligand for integrin, a transmembrane protein essential for cell-extracellular matrix (ECM) adhesion [126,127,128]. Several tissue engineering studies have demonstrated that RGD modification is beneficial for cell adhesion and proliferation.

### 3.3. Protein Polymer-Based Bioinks

Protein polymer-based bioinks include collagen, fibrin, and gelatin. Since most of these are made from animal tissues, they contain ECM molecules that directly interact with cell membrane proteins, which produces high biocompatibility. Collagen accounts for the majority of ECM components in the body and is widely used in biological experiments. Collagen is acid-soluble and self assembles when the temperature and pH are adjusted to physiological levels. For more powerful crosslinking, additional crosslinking agents such as n-hydroxysuccinimide and 1-ethyl-3-3-(3-dimethylaminopropyl)carbodiimide are sometimes used.

Fibrin is formed when fibrinogen present in the blood undergoes a rapid reaction with thrombin to induce clotting after a blood vessel is injured. Rapid crosslinking is useful for obtaining high shape fidelity by rapidly changing the ejected bioink to a solid during 3D cell printing. Hinton et al. produced a gelatin slurry media containing thrombin and printed an alginate hydrogel in which fibrinogen and calcium were mixed [97]. The alginate containing fibrinogen was polymerized by thrombin in the gelatin slurry as soon as it was discharged from the printing nozzle to support calcium-mediated solidification of the alginate.

Gelatin is made by denaturing collagen in the skin and bone tissues of animals and can be mass-produced inexpensively. Gelatin is a liquid at a temperature of 40 °C or higher, and reversible gelation proceeds through random coil formation below 30 °C. This property allows gelatin to exist as a liquid at physiological temperatures. Some researchers have devised a method of printing a cell monolayer by printing the gelatin bioink encapsulating the cells by extrusion and then washing the liquefied gelatin at a physiological temperature [94,129]. On the other hand, to irreversibly crosslink gelatin, gelatin-methacrylate (GelMA) bioinks capable of UV photopolymerization are produced by methacrylating gelatin, making it possible to maintain the printed structure even at a physiological temperature [130,131].

### 3.4. Decellularized Extracellular Matrix-Based Bioinks

Materials obtained by decellularizing animal tissue (dECM) are considered a promising material for bioink for 3D cell printing. dECM bioinks are better able to reproduce the inherent complexity of the original ECM organization. When the original tissue is decellularized, it is possible to preserve the intrinsic composition of tissue-specific proteins, proteoglycans, and glycoproteins, which is difficult to engineer through traditional bioink manufacturing methods [132,133]. Because dECM bioink retains collagen components present in vivo, self-assembly occurs under physiological conditions, similar to collagen. Pati et al. first proposed a method of using dECM bioink to print target tissues [134]. The dECM bioink derived from the target tissue promoted the differentiation of cells with the unique lineage of the tissue that matched the target tissue rather than the existing collagen bioink. Han et al. also reported the tissue-specific functionalities of different kinds of tissue/organ-derived dECM bioinks by demonstrating the specific tissue-related behaviors in multipotent adult stem cells encapsulated in each dECM bioink [135]. Studies on the efficacy of heart, skeletal muscle, liver, fat, skin and corneal dECM bioinks have been extensively reported [93,120,134,136,137,138]. The physical properties of crosslinked dECM bioinks are weaker than that of the actual tissue. To overcome this limitation, Jang et al. reported a method of increasing the strength of printed tissue made of heart dECM bioink by implementing vitamin B2 (0.01% w/v)-induced UVA crosslinking [121].

## 4. Therapeutic Applications

A longer life span increases the likelihood of several age-related disorders. Most of these disorders are related to cartilage, cornea, muscle, and vasculature. Tissue/organ transplantation is the best option for curing lesions and defects of these tissues/organs. However, alternative solutions are needed to overcome the problem of worldwide donor shortages.

Cell-based therapy provides a new paradigm in regenerative medicine since it has the potential to replace the patient’s tissue as well as their remedial paracrine effects [139,140]. However, cell-based therapeutics face the challenges of low survivability and differentiation potential of transplanted cells. A tissue engineering approach, based on the combination of cells, biomaterials, and biochemicals, may be able to overcome these limitations [141,142]. As such, 3D cell printing is a powerful technology for constructing tissue-engineered tissue/organs. Moreover, 3D patterning of cells and biomolecules enables the fabrication of complex structures of customized sizes that mimic native organs [143]. Many researchers have investigated the therapeutic application of 3D cell printing for regenerative medicine. This section describes the recent developments in 3D cell printing of tissue-engineered constructs for therapeutic purposes.

### 4.1. Cartilage Regeneration

**Figure 2:**
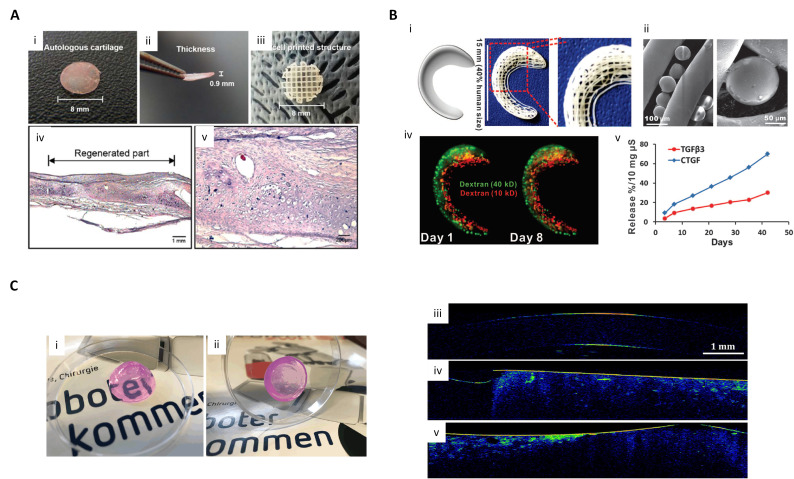
Therapeutic applications of 3D printing for cartilage and cornea regeneration. (**A**) 3D cell printed structure for cartilage defect treatment. (i,ii) Autologous cartilage; (iii) Cell-printed structure; (iv,v) HE staining result of in vivo cell-printed structure implantation (reproduced with permission from ref [144]; copyright 2017 Wiley-VCH). (**B**) Scaffold spatiotemporally releasing growth factor for meniscus defect treatment. (i) Anatomic reconstruction of a human meniscus and 3D-printed meniscus scaffold; (ii) PLGA microspheres encapsulating growth factors with PCL microfibers; (iii) Maintenance of fluorescent dextrans in meniscus scaffold from Day 1 to Day 8; (iv) growth factor release profile from the scaffold over time (reproduced with permission from ref [147]; copyright 2014 American Association for the Advancement Science). (**C**) 3D structure of bioprinted models with human corneal stromal keratocytes and their optical properties. (i,ii) Transparent bioprinted dome-shaped artificial corneas consisting of 0.5% agarose and 0.2% collagen Type I. (iii—v) Optical coherence tomography images of native rabbit corneas; (iii) Cell-free blend of 0.5% agarose with 0.2% collagen Type I; (iv) Corneal stromal keratocyte-loaded hydrogel blend; (v) corneal stromal keratocyte-loaded hydrogel blend (reproduced with permission from ref [148]; copyright 2019 Wiley-VCH).

Previous clinical treatments of articular cartilage damage have included microfracture and autologous cartilage transplantation. However, these techniques often result in negative fibrocartilage formation. To overcome the existing treatment limitations, researchers have been conducting research on implantable cartilage substitutes using 3D cell printing technology. Kundu et al. produced a hybrid-type cartilage substitute structure composed of chondrocyte, alginate, and PCL [111]. Park et al. developed an autologous cartilage structure composed of autologous chondrocyte, alginate, and PCL using 3D cell printing for auricular construction (Figure 2A). PCL was used for the long-term stability of the implanted structure. In vivo evaluation showed the excellent cartilage tissue regeneration, but the stiffness of PCL caused the abrasion of cartilage tissue around the graft site [144]. Rathan et al. functionalized alginate with cartilage dECM to use it as a bioink, since cartilage dECM can increase the cell compatibility and chondrogenic potential of alginate bioink. The bioink encapsulating mesenchymal stem cells (MSCs) and TFG-β3 offered robust chondrogenesis in vitro. Additionally, a hybrid structure with a 3D printed PCL network and cell-laden dECM-functionalized alginate bioink achieved a compressive modulus comparable to native cartilage tissue while retaining cell viability [145]. Hung et al. developed a water-soluble biodegradable polyurethane (PU) as a bioink to produce a 3D printed cartilage structure with a high strain recovery capability. Since the PU bioink was soluble in water, it could be mixed with bioactive molecules such as hyaluronic acid or growth factors. Four weeks after the printed structure was implanted at the site of osteochondral defects in rabbits, safarin-O staining showed that a large amount of glycosaminoglycan (GAG) was secreted, indicating a high production of cartilage [146].

Research has also been conducted to replicate the multi-zonal organization of cartilage. Meniscus consists of a white zone and red zone; the white region found in the inner zone of the meniscus is composed of chondrocyte-like cells with abundant GAG and type II collagen, while the red zone, found in another region of the meniscus, has collage type I fibroblast cells. Taking into account these spatial characteristics, Lee et al. designed a scaffold to treat meniscus damage. They placed transforming growth factor β3 (TGFβ3) in the white zone of the scaffold and connective tissue growth factor (CTGF) in the red zone. The released growth factors induced zone-specific differentiation of human synovium MSCs, followed by the formation of zone-specific matrix. Furthermore, results showed that zone-specific phenotypes occurred three months after implantation in a sheep meniscectomy model (Figure 2B) [147].

### 4.2. Cornea Regeneration

Damage to the cornea, the outer layer of the eye that refracts light, can cause blindness. Although there are synthetic substitutes for treating corneal damage, they may induce an immune response due to a lack of biocompatibility. Therefore, when constructing a structure for corneal treatment, transparency and biocompatibility should be key criteria. Duarte et al. produced a dome-shaped structure similar to the actual cornea by printing keratocytes using a mixture of agarose and collagen as bioink. The printed cells were viable and showed similar characteristics to native keratocytes, and the fabricated structure showed similar transparency to an actual cornea (Figure 2C) [148]. The orthogonal arrangement of the lamellae of the cornea plays an important role in determining the transparency of the cornea. Kim et al. implemented a cornea-specific lamellae structure using 3D cell printing. Keratocytes encapsulated in cornea dECM bioinks were printed and the nozzle diameter was optimized to allow cell alignment during printing. Using this optimized process, the nozzle movement direction was vertical when printing the lower layer and upper layer. As a result, cells and collagen fibers aligned in a lattice pattern similar to the structure of the native cornea. Four weeks after transplantation of the 3D printed structures to rabbit corneas, the 3D printed group showed better transparency than the non-printed group [149]. The cornea is composed of an epithelial layer and a stromal layer. Sorkio et al. reported a fabrication method for a 3D cornea-mimicking structure layered with a stromal layer and an epithelial layer using laser-assisted bioprinting (LaBP). A laminin and collagen I mixture was used for the base of the bioink. In this study, human embryonic stem cell-derived limbal epithelial stem cells (hESC-LESCs) were used for the epithelial part of the print and human adipose-derived stem cells (hASCs) were selected for the fabrication of the stromal part [150].

### 4.3. Skeletal and Cardiac Muscle Regeneration

**Figure 3:**
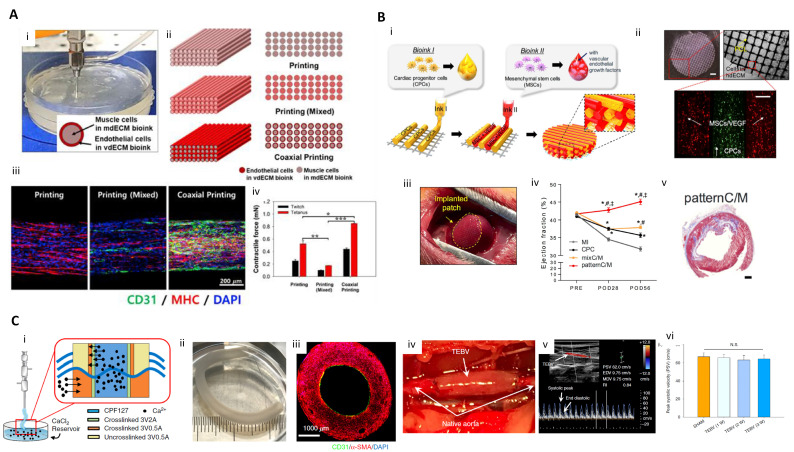
Therapeutic applications of 3D printing for skeletal muscle, cardiac muscle and vasculature regeneration. (**A**) 3D cell printed pre-vascularized muscle construct. (i) 3D cell printing pre-vascularized muscle constructs through a coaxial nozzle; (ii) Schematic illustration of the printing, printing (mixed), and coaxial printing groups. Overall views of the constructs (left). Cross-sectional views of the constructs (right); (iii) IF images of the different muscle constructs; (iv) Twitch and tetanus forces of the printing, printing (mixed), and coaxial printing groups (n = 5); * *p* < 0.05, ** *p* < 0.01, and *** *p* < 0.001 (reproduced with permission from ref [100]; copyright 2019 Elsevier). (**B**) 3D printed complex tissue construct for cardiac repair. (i) Illustration of pre-vascularized stem cell patch including multiple cell-laden bioinks and supporting PCL polymer; (ii) Fabricated patch including the two types of cell-laden bioink and PCL supporting layer (Scale bar (top left), 1 mm; Scale bar (bottom), 200 μm); (iii) Photo of implanted patch; (iv) Ejection fraction values at baseline and after 4 and 8 weeks. Error bars represent sem (* *p* < 0.05 compared with MI; # *p* < 0.05 compared with CPC; ‡ *p* < 0.05 compared with mixC/M); (v) Masson’s trichrome staining results of a whole heart for a pattern CPC/MSC group (scale bars, 1mm) (reproduced with permission from ref [151]; copyright 2017 Elsevier). (**C**) Triple-coaxial cell printing-based vascular graft containing endothelium and smooth muscle. (i) A schematic of reservoir-assisted triple-coaxial cell printing; (ii) Triple coaxial cell-printed tissue-engineered blood vessels (TEBVs) with inner diameter of 2 mm and WT of 1 mm; (iii) Cross-sectional view of the vessel including a thin endothelial cell layer and a thick smooth muscle cell layer stained with CD31 and alpha-SMC, respectively; (iv) implanted TEBV as interposition grafts; (v) The flow pulse of the TEBVs showing a regular spectrum composed of rapid-sharp systolic peaks and slow, flat end-diastolic velocity; (vi) the peak systolic velocity of TEBVs showing no significant differences for up to 3 weeks compared to that of normal rats (sham control, N.S: not significant) (reproduced with permission from ref [105]; copyright 2019 AIP Publishing).

Both skeletal muscle and myocardium have sarcomeres and exhibit strong contractile properties through highly organized bundle structures. Skeletal muscle contains bundle structures arranged in parallel, and regenerating the contractile bundle structure of these skeletal muscles is important for the treatment of skeletal muscle loss. Kang et al. used a mixture of gelatin, fibrinogen, and hyaluronic acid as a bioink to encapsulate C2C12 myoblasts to print muscle structures. For cell arrangement, a uni-axial constraint was generated through tissue shrinkage caused by cell-mediated ECM remodeling. After the structure was cultured for seven days after printing, it was confirmed that the muscle cells were aligned in one direction in the 3D muscle structure. This mature muscle structure was transplanted ectopically and subcutaneously into nude rats and embedded with the common fibular nerve (CPN) for integration with the nerve. After two weeks, the nerve and muscle fibers were in contact, and the muscle function was improved [26]. Choi et al. printed a pre-vascularized muscle structure imitating the hierarchical architecture of vascularized muscle using the coaxial nozzle printing technique. Muscle cells encapsulated in skeletal muscle dECM bioink was ejected through the core nozzle. Vascular endothelial cells were encapsulated in vascular dECM bioink and extruded through the shell nozzle. When the 3D-printed muscle structures were implanted in a rat model of volumetric muscle loss, vascularization, innervation, and muscle contraction recovery were improved in the coaxial nozzle printing group compared to other groups. This study showed that an implant structure with spatially controlled, tissue-specific bioink and cells provides organized microenvironmental cues to differentiate each cell type, leading to more effective muscle regeneration (Figure 3A) [100].

Myocardium has a shorter, branched bundle structure compared to skeletal muscle and is involved in involuntary movement. Gaetani et al. printed human fetal cardiac myocardial progenitor cells encapsulated in a hyaluronic acid/gelatin-based bioink to fabricate heart patches. In this study, a porous structure was fabricated using 3D cell printing to facilitate the supply of oxygen and nutrients. The porous patch showed higher cell viability of human fetal cardiomyocyte progenitor cells compared to the group with a non-porous structure. The patch maintained its cardiogenic phenotype for up to 1 month after printing and was successfully transplanted into a mouse myocardial infarction (MI) model. The results showed decreased cardiovascular hypertrophy and fibrosis and improved myocardial viability [152,153]. Jang et al. spatially patterned human c-kit+ cardiac progenitor cells (hCPC) and hTMSC with vascular endothelial growth factor (VEGF) using heart dECM bioink and an extrusion-based cell printing system to print a patch for treating the mouse MI model. After transplantation, the patterned patch promoted vascularization more effectively than the non-patterned patch, alleviated left-ventricle remodeling, and improved myocardial function (Figure 3B) [151]. A study by Wang et al. fabricated centimeter-scale cardiac tissue equivalents. For the scalability of tissue-engineered constructs, the diffusion of oxygen and nutrients is an important consideration. Accordingly, they alternately printed sacrificial bioink made of gelatin and cardiomyocytes-laden fibrin-based composite bioink, enabling the inflow of cell culture media to the inside part of the construct. During cultivation, PCL frame-induced tension facilitated cardiomyocyte alignment with sarcomere structure [154].

### 4.4. Vasculature Regeneration

Synthetic polymer-based vascular substitutes (e.g., Dacron and Teflon) have been used to treat ischemic cardiovascular disease; however, side effects such as acute thrombosis, hyperplasia, and aneurysm occurred when the vascular substitutes had a diameter of 6 mm or less [155,156]. This is because the relatively small diameter results in a lower blood flow rate and increases the irregular interaction between the synthetic polymer and the blood. Therefore, vascular substitutes for treating ischemic cardiovascular disease must be capable of withstanding hemodynamic stress and endothelium formation that inhibits thrombosis.

To meet these requirements, researchers are developing a tissue-engineered blood vessel that mimics native tissue architecture. Xu et al. printed an endothelial cell and smooth muscle cell bilayered tubular structure using GelMa bioink and an extrusion bioprinting system. However, endothelium formation was not achieved with this printing strategy [157]. Gao et al. optimized the ratio of vascular dECM bioink to alginate hydrogel to optimize the cell behavior of endothelial cells and smooth muscle cells. This study used a tri-axial nozzle for blood vessel printing. In the core nozzle, Pluornic F-127 containing calcium ions was extruded. Endothelial cells were released by the middle nozzle and smooth muscle cells were discharged through the shell nozzle. After printing, the blood vessel was matured until endothelium formation occurred. Three weeks after the 3D-printed blood vessel was implanted in rat abdominal aortas, it showed high patency, intact endothelium, remodeled smooth muscle, and integration with host tissues (Figure 3C) [105].

In many cases, ischemic disease is not limited to large arteries and additional methods are required to enhance microvascular perfusion. T. Mirabella et al. printed a patterned channel using sugar as a sacrificial bioink and embedded the printed channel network on a fibrin gel. After removing the sugar, endothelial cells were seeded in the empty space to prepare a patch with endothelial cell-lined lumen. The patch was implanted into rodent models of hind limb ischemia and MI. The vascular graft restored distal tissue perfusion, which prevented capillary loss, muscle atrophy, and loss of function [158].

## 5. Drug Screening Applications

Animal models are widely used to test drugs. However, differences in physiology, pharmacokinetics, and genetics reduce the reliability of animal models [159]. Although humans and mice share the same genes, their regulation mechanism is different [160]. Drug testing results vary according to which species is used [161,162]. Therefore, there is a significant demand for models made of human cells as an alternative to animal models. Furthermore, if patient-derived cells are used to produce drug-testing models, it is possible to produce personalized medicine. To fabricate a physiologically relevant model, researchers have focused on recreating complex architecture and cell-cell interactions [163,164]. Thus, 3D cell printing is a promising technology for achieving these objectives.

Because the liver and kidney are susceptible to drug-induced injury, drug toxicity is a crucial consideration when developing new drugs. To produce drug toxicity testing platforms, there have been several attempts to replicate the complex tubular structures of the liver and kidney using 3D cell printing technology. In addition, due to the restrictions on animal testing in the cosmetic industry, in vitro skin models have been developed relatively earlier than other tissues. The utility of 3D cell printing for the fabrication of full-thickness skin models has been demonstrated by several studies. 3D cell printing also has the advantages of cost-effectiveness and the ability to customize production. These characteristics have synergistic effects with the use of patient-derived cells to personalize medicine. For example, 3D-printed cancer models have been developed to create personalized disease models for patient-specific drug efficacy tests.

### 5.1. Liver Model

The liver is a major organ that regulates the overall metabolism in the body and plays a crucial role in pharmacokinetics/pharmacodynamics (PK/PD) of drugs by taking charge of biotransformation [165]. This makes hepatotoxicity a critical safety issue to consider in drug development. Therefore, it is essential to develop an in vitro hepatic model with the functional maturity to precisely simulate and anticipate the behavior of various substances in the liver. Many researchers have applied 3D printing technology to produce mature hepatic functions by mimicking a hexagonal lobule and hepatic sinusoidal structure where the hepatocyte and blood vessel are aligned in a specific manner. For example, Bhise et al. used 3D printing technology to create an in vitro hepatic model using a photocurable GelMA bioink [122]. In the study, hepatic spheroids encapsulated in GelMA were printed and cultured on a PDMS-based bioreactor with a hexagonal shape and continuous perfusion. Hepatic markers such as albumin, A1AT, and transferrin were maintained for 4 weeks, and significant hepatic toxicity was observed in acetaminophen treatment for 6 days, indicating its feasibility as a drug testing platform.

**Figure 4:**
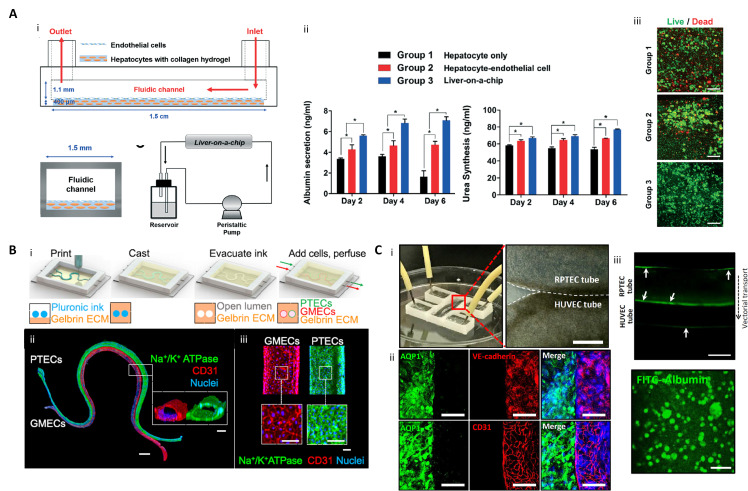
3D printed liver-on-a-chip and kidney-on-a-chip. (**A**) 3D printing-based liver-on-a-chip (i) Schematic diagram of one-step fabricated liver-on-a-chip with a vascular channel; (ii) Liver function analysis with albumin and urea tests (* *p* < 0.05); (iii) hepatocyte viability on Day 6 (scale bar: 100 μm) (reproduced with permission from ref [94]; published by The Royal Society of Chemistry). (**B**) 3D vascularized proximal tubule on a chip (3D VaPT) model; (i) Fabrication process of the 3D VaPT model; (ii) The fluorescence image of the 3D VaPT model. PTECs are presented in blue, GMECs in red, and nuclei in blue (Scale bar: 1 mm). The cross-sectional views of each microchannel (scale bar: 100 μm); (iii) Magnification of PTECs and GMECs channel in the 3D VaPT model (scale bar: 100 μm). (**C**) 3D cell-printed renal proximal tubule-on-a-chip with tubular RTPEC and HUVEC constructs. (i) Microfluidic system and dual tubular constructs of the chip after fabrication; (ii) Fluorescence image of renal proximal tubular markers (AQP1, aquaporin 1), and vascular markers (vascular endothelial (VE)-cadherin, and CD31) in the chip after maturation. The renal proximal tubular marker is presented in green, vascular endothelial markers are red, and nuclei are blue (scale bar: 100 μm); (iii) Albumin reabsorption between RTPEC and HUVEC tube via vectorial transport. Arrows indicate the intracellular accumulating albumin within the RPTEC and sidewall of the vessel (scale bar: 400 μm) (reproduced with permission from ref [123]; copyright 2020 Elsevier).

Taking into account the multi-cellular components and microstructure of the liver, Lee et al. developed a one-step 3D-printed liver-on-a-chip consisting of compartmentalized parts of a hepatocyte and vessel and a PCL chip body [94]. The vascular part of the monolayer was achieved by printing the endothelial cells encapsulated in gelatin bioink, and the hepatic sinusoid-mimicking channel was produced. In the hepatic model with a monolayer vascular portion and culture media perfusion through the microchannel, the viability and synthesis of albumin and urea were significantly improved (Figure 4A).

While the previous study was a pump-based chip, the same research group reported a liver-on-a-chip with a biochemical microenvironment and biliary system [166]. In the chip, liver dECM bioink and a hepatic progenitor cancer cell line were used for the biochemical environment and biliary structure, respectively, and pumpless fluidics were used for more convenient operation. In the experimental group with a system for washing bile acid that causes hepatic toxicity, the expression of liver functional markers such as albumin, AFT, and TTR was upregulated, and the expression of CYPs related to drug metabolism increased. Moreover, the drug sensitivity using acetaminophen increased, as in the native liver, compared to the conventional 2D model.

### 5.2. Kidney Model

Although some drugs used for medical treatment can cause nephrotoxicity, there are many cases where they are inevitably used [167]. In addition, renal toxicity of drug candidates is found relatively later in the drug development process, leading to an increase in the cost of developing new drugs [168]. Therefore, a human renal tissue model is urgently required. Researchers developing renal tissue in vitro have produced complicated and perfusable renal tubular constructs using 3D printing technology. The kidney is made up of a functional unit called a nephron, where tubular structures such as proximal tubules, glomeruli, and blood vessels are compartmentalized and interact closely, and 3D printing technology has enabled the replication of such complicated renal structures.

LIN, Neil YC, et al. developed a 3D vascularized proximal tubule model with a perfusable proximal tubule and blood vessel constructs [169]. First, a dual-microchannel portion was printed with a fugitive bioink composed of Pluronic F127 and high-molecular-weight poly(ethylene oxide). Then, a hydrogel composed of gelatin and fibrinogen was poured and crosslinked. After that, the renal proximal tubular epithelial cells (RPTECs) and glomerular microvascular endothelial cells were seeded in each microchannel produced by removing the fugitive bioinks. These matured into a vascularized renal tubular model capable of perfusion. This construct enabled substance exchange, such as albumin reabsorption and glucose reabsorption, between the proximal tubule and blood vessels, which was not possible with conventional models (Figure 4B).

Singh et al. developed a vascularized kidney-on-a-chip consisting of a renal proximal tubule and blood vessels. This was achieved using direct, coaxial cell-printing and a hybrid bioink made of kidney dECM and alginate [123]. The hybrid bioink improved the viability and the expression of functional markers such as gamma-glutamyl transpeptidase (GGT), aquaporin 1 (AQP1), and kidney-specific cadherin (KSP) in RPTECs compared to using medical-grade collagen type I bioink. In addition, the expression of functional markers in the proximal tubule was upregulated compared to the conventional 2D and 3D culture methods, indicating that the tubular architecture was also a specialized cue for functional maturation of the renal proximal tubule. A vascularized renal proximal tubule-on-a-chip was fabricated by printing the proximal and vascular tubes close together but compartmentalized on the chip body made of PCL. When the culture media was perfused in each tube, receptor-mediated endocytosis of FITC-albumin was observed, confirming that native-like renal behavior was achieved (Figure 4C).

### 5.3. Skin Model

The European Union’s ban on animal testing for cosmetic products has accelerated the development of in vitro skin models to replace animal testing. The skin consists of dermis, epidermis, and hypodermis, and plays an important role in temperature regulation, tactile sensing, and acting as a physical barrier. It also contains a vascular network, nerves, and appendages such as hair, glands, and nails. To develop cosmetics and drugs to be applied to skin, in vitro skin models must mimic the complexity of native skin, and 3D cell printing technology can be utilized to replicate this complex 3D anatomy.

**Figure 5:**
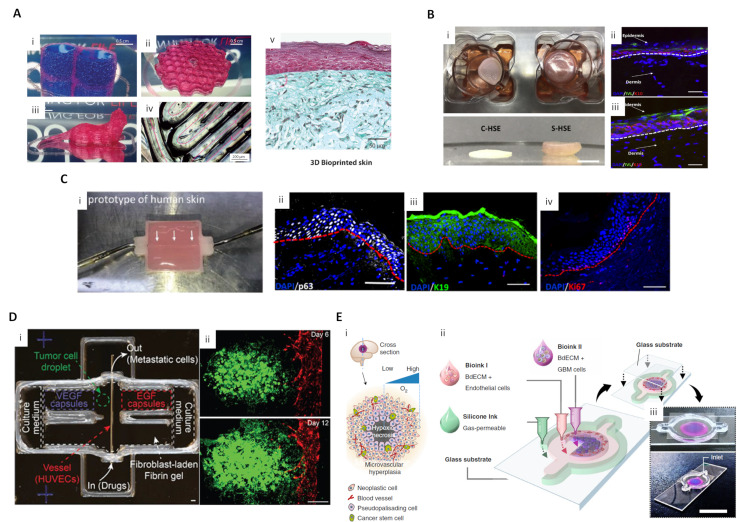
3D-printed skin and cancer model for drug screening applications (**A**) Diverse-shaped 3D-printed structures using a scaffold-free approach with gelatin-alginate-fibrinogen bioink and 3D-printed skin. (i) A water-tight structure with two compartments filled with blue dyed liquid; (ii) A complex structure with hollow honeycomb features of 200 μm width; (iii) A centimeter-sized complex object with overhanging structures; (iv) A closer view of a print showing 200 μm wide printing lines. Pink arrows show the printing movement of the nozzle; (v) Optical microscopy of Masson’s Trichrome images of bioprinted skin after 26 d of culture (reproduced with permission from ref [170]; copyright 2017 Wiley-VCH). (**B**) 3D-printed skin using skin dECM bioink. (i) Representative photographs of 3D cell-printed in vitro skin equivalents using type I collagen (C-HSE) and skin-derived bioink (S-HSE); (ii, iii) Expression of epidermal differentiation markers with C-HSE (ii) and S-HSE (iii) on Day 10 after ALI culture (reproduced with permission from ref [120]; copyright 2018 Elsevier). (**C**) Perfusable vascularized skin equivalent composed of epidermis, dermis, and hypodermis. (i) A prototype of the fabricated perfusable full-thickness skin construct, (ii) immunostaining of p63 (white), (iii) K19 (green), and (iv) Ki67 (red) in the skin construct (reproduced with permission from Ref [171]; copyright 2019 Wiley-VCH). (**D**) 3D-printed in vitro metastatic model. (i) Photo of a 3D-printed culture chamber to test guided tumor cell dissemination; (ii) Fluorescence images of a metastatic model on Days 6 and 12, showing that A549s approach and enter the vasculature through the fibroblast-laden fibrin gel (green channel: GFP-expressing A549s, red channel: RFP-expressing HUVECs) (reproduced with permission from ref [172]; copyright 2019 Wiley-VCH). (**E**) (i) Schematic illustration of a cross-sectional view of a native GBM; (ii) Schematic illustration of the process for printing the GBM-on-a-chip with various bioinks and other materials to construct a compartmentalized structure; (iii) Photographs of a mock GBM-on-a-chip (reproduced with permission from Ref [124]; copyright 2019 Nature Publishing Group).

Pourchet, Léa J., et al. produced dermis analogs by printing human dermal fibroblasts using a mixture of gelatin, alginate, and fibrinogen as a bioink, and seeding human epidermal keratinocytes on a dermis structure to create a skin model. After 26 days of culture, the morphology of the mature skin model was similar to that of human skin (Figure 5A) [170]. ECM plays an important role in enhancing cell-cell interaction, and cell-ECM interactions are important for developing epidermal organization. Accordingly, to promote cell activity and their interaction, Kim et al. used skin dECM bioink that produces a native-like microenvironment to print an in vitro dermal/epidermal skin model. Fibroblasts in skin dECM bioink showed better cell behavior for skin regeneration than collagen type 1 bioink. The skin dECM bioink produced less contraction of the dermis structure that occurs during the maturation process. In contrast to collagen type 1 bioink, this characteristic improved the epidermal organization. The prevention of tissue contraction was due to the presence of thick collagen fibers and ECM components, such as elastin and hyaluronic acid, in the skin dECM bioink. This skin dECM bioink enabled the fabrication of an in vitro model that is closer to the native skin in terms of its barrier function compared to collagen type 1 bioink (Figure 5B) [120].

The same research group developed a 3D-printed skin model, based on skin dECM bioink, with perfused blood vessels in the dermis and hypodermis sections. A PCL-based transwell platform was suggested to integrate the perfusable channel between the dermal and hypodermal compartments, and the gelatin-based vascular bioink was used to print a vessel-like channel with an endothelial cell monolayer. The maturation of this skin model was structurally assessed using functional markers representing the epidermis (stratified structure), dermis (dermal-epidermal junction, secreted ECM composition), hypodermis (lipid droplets), and vascular channels (endothelium). These experiments demonstrated a successful maturation of skin tissue. Moreover, histological analysis of a skin stemness marker showed that the skin model containing vascularized hypodermis and dermis shared more structural similarities with native human skin than the control group (Figure 5C) [171].

In addition to keratinocytes, skin epidermis also contains melanocytes, which are critical for skin pigmentation. Traditional methods of pigmented 3D skin fabrication are limited in their ability to produce uniform skin pigmentation. Ng et al. demonstrated the advantage of 3D cell printing to overcome this limitation. Using an inkjet printing system, the dermal portion was printed with fibroblast-laden collagen bioink, and keratinocytes and melanocytes were printed on the dermal construct. Compared to the conventional manual-casting approach, the 3D-printed pigmented skin construct had greater similarity to native skin tissue in the presence of well-developed stratified epidermal layers and the presence of a permanent basement membrane layer [173].

### 5.4. Cancer Model

Cancer is among the leading causes of death worldwide, and differences between in vitro and in vivo efficacy is still a major limitation in the development of effective drugs and therapies [174]. Therefore, advanced cancer treatment will require a clinically applicable model. Furthermore, many studies have been conducted on the toxicity of anticancer drugs, making this a high-priority area of development for pharmaceuticals. Cancer growth and progression are affected substantially by complex microenvironments, including multiple cell types, different types of ECM molecules, and their interactions. 3D bioprinting techniques have been utilized to create tumor micro-environments because of their advantages in this application such as precise composition and well-organized spatial distribution of tumor cells and extracellular components.

Meng et al. developed 3D metastatic in vitro printed models with a precisely controlled spatial positioning of cells, programmable growth factor release capsules, and biomaterials. The growth factor gradient was controlled using programmable release capsules triggered by laser irradiation to mimic tumor metastasis and angiogenesis. After guided migration of tumor cells and endothelial cells, the efficacy of immunotoxin was tested to demonstrate the utility of the 3D vascularized cancer model as a drug screening platform (Figure 5D) [172].

Furthermore, 3D cell printing technology also showed the potential to simulate multi-tissue/organ metastasis since it enables the deposition of different tissues in one platform. Cui et al. simulated breast cancer metastasis to bone tissue using an SLA 3D printing system with bioink optimized for cancer cells, endothelial cells, and osteoblasts. This system enabled the observation of transendothelial migration and colonization of cancer cells in vitro [175]. Yi et al. 3D printed glioblastoma-on-a chip to study patient-specific responses to chemoradiotherapy. Brain dECM bioink was used to encapsulate patient-derived tumor cells and endothelial cells and print a compartmentalized cancer-stroma concentric-ring structure. The printed cancer model sustains a physiologically relevant radial oxygen gradient by combining a 3D-printed gas-permeable silicon wall and a glass coverslip. This model effectively mimicked the architectural, biophysical, and biochemical characteristics of natural glioblastoma tumors. The study also observed patient-specific resistance to temozolomide and chemoradiation therapy and patient-specific sensitivity to potential combinations of anti-cancer drugs (Figure 5E) [124].

## 6. Conclusions and Future Perspectives

In conclusion, 3D cell printing techniques have demonstrated a promising ability to fabricate human organs. With the advancement of control systems and the introduction of new bioinks, the techniques have evolved from the fabrication of polymer-based scaffolds to direct cell printing. These developments have marked a shift toward realistic tissue/organ fabrication for therapeutic and drug screening platforms. Despite these achievements, there are several limitations that still need to be addressed.

The first challenge is to 3D print cell constructs at the macro scale. While many types of 3D-printed cell constructs have been reported, the size of these constructs is still much smaller than actual human organs [42]. The fabrication of large-volume cell-printed constructs requires cell viability, structure stability, and molecular transportation. Although most of the printing methods and bioinks in this review showed over 80% cell viability, the process of printing has the risk for cell damage such as dehydration [51,176]. Since the fabrication of large-volume constructs is time-consuming, the viability of the cell may decrease over longer printing times. This factor could be addressed by using a closed chamber system with a humid environment and a low printing temperature, which protects cell viability during a lengthy printing process [87,95].

Molecular diffusion is another limitation that should be considered in future studies. In the human body, oxygen and nutrients are supplied, and molecular wastes such as carbon dioxide are removed through the blood in the capillaries. This process is governed by diffusion. The capillaries form a network for stable transportation to prevent spatial limitations of diffusion. This ensures stable diffusion despite the large scale of tissue structures in the human body. Without taking into account the limitations of diffusion, printed constructs may not fully mimic their natural function due to an insufficient supply of oxygen and nutrients [177,178]. Porous structures formed by a polymeric framework has been used to promote oxygen and nutrient supply [26,179]. The porous structure allows the flow of culture medium to maintain the viability and functionality of the cells in a large-volume structure. Although this strategy enables a relatively large-volume structure compared to the conventional lab-on-a-chip platform, the fabricated structures are insufficient for transplantation.

Another challenge to overcome is fabricating tissues/organs with multiple cell types. Although 3D cell printing techniques have a precise control system for the location of the cells, further research is needed on how to induce differentiation and provide optimal culture conditions for co-cultures. Despite several molecules, specialized culture mediums, and bioinks have been reported, no single or combination of these factors are enough to maintain microenvironments for multiple types of cells [180,181]. Tissue-specific bioink is one potential method to replicate the appropriate microenvironment [134]. dECM could provide the optimal composition of the extracellular matrix (ECM), which promotes maturation and differentiation of cells. Therefore, the combination of tissue-specific bioink, 3D cell printing systems, and tissue-mimetic design could replicate the proper structure and chemical microenvironment to recreate the natural function of tissue/organs.

While the development of new printing techniques and bioinks provides advanced tissue/organ constructs, a multi-organ system should also be considered. Since the organs in the human body are connected and interact with each other, realistic functionality cannot be achieved without this interconnected system [94,182,183]. A conventional machining process and MEMs-based multi-organ-on-a-chip was reported and showed enhanced functionality and suitability for pharmacology studies [184,185,186]. However, there have not been studies on 3D cell printing-based multi-organ-on-a-chip. Since organs are connected through the blood vessels, fabrication of a vascular network is a key factor for a multi-organ system. For this objective, current research has used sacrificial bioink to fabricate the hollow structure, and then endothelial cells are seeded on the wall to form the endothelial layer [92,96]. The pre-vascularized muscle construct was fabricated using a coaxial nozzle to print muscle and endothelium as the core and shell structure, respectively [100].

Personalized medicine is also a growing area of interest. Since there are different factors related to genetics and physical environments between patients, strategies for treatment and prevention should be different for individual patients [187,188,189]. The introduction of induced pluripotent stem cells (iPSC) led to tissue models incorporating the patient’s pathophysiology information such as genetic mutation and functional abnormality [190,191,192]. However, conventional cell cultivation methods, including 2D and 3D methods, are insufficient to recreate the complex functionality of natural tissue/organs. The combination of patient-derived iPSC, 3D cell printing techniques, and advanced bioinks may facilitate the fabrication of patient-specific tissue/organs, which could be used as a testing platform for new drug development.

Research on 3D cell printing techniques has demonstrated their ability to fabricate complex 3D constructs. The development of cell printing methods, and bioinks provide new direction in regeneration medicine and drug development. Even though there are several limitations to be considered, the technique is being improved by a multidisciplinary approach, and fabricate realistic tissue/organs are expected to be available for clinical and pharmaceutical industry in the near future.

## Figures and Tables

**Table 1:** Comparison of 3D cell printing techniques.

Printing Methods				
Category	LIFT	SLA	Inkjet	Extrusion
Cost	High	High	Low	Medium
Resolution	40 μm	100 nm	20 μm	100 μm
Speed	200~1600 mm/s	10 μm~20 mm/s	10,000 droplets/s	10 μm~50 mm/s
Bioink Viscosity	1–300 mPa s	~5 Pa s	3–12 mPa s	~600 kPa s
Gelation mechanism	Photo	Photo	Chemical, Photo	Thermal, Chemical, Photo
References	[24,37,38]	[39,40,41]	[20,24,42]	[24,40,43]

**Table 2:** Comparison of synthetic polymer, carbohydrate polymer, and protein polymer for bioink.

Source	Type	Printing Method	Gelation Method	Cell Viability	Advantages (A) & Disadvantages (D)	Ref
Synthetic polymer	PEGDA	SLA	Photo	>95%	A: High transparency and tunable mechanical properties D: Potential cytotoxicity caused by UV irradiation, low cellular adhesiveness, and cell proliferation	[39,109]
	PEGTA	SLA	Photo	n/a	A: Better rheological properties and cell response than PEGDA	[110]
Carbohydrate polymer	Alginate	Extrusion	Ionic	>85%	A: Low cost and rapid gelation D: Low cellular adhesiveness and limited cell proliferation and interaction	[111]
	Agarose-based	Extrusion	Thermal	n/a	A: High mechanical strength, low price, and high shape integrity DA: Inferior cell adhesion	[112,113]
	Gellan gum & GelMa	Extrusion	Ionic	n/a	A: Strong mechanical properties, high printability D: Slow gelation and low cell survival	[114]
Protein Polymer	Collagen	Inkjet, laser, extrusion	Thermal	>92%	A: High cellular adhesiveness and promotion of cell migration and proliferation D: Insufficient mechanical properties for structural support due to slow gelation	[47,94,115,116,117]
	GelMA	SLA	Photo	>80%	A: Can moderate mechanical properties for structural support, high cellular adhesiveness, and promotion of cell spreading and proliferation D: Potential cytotoxicity caused by UV irradiation and low mechanical properties	[58]
	Fibrin	Inkjet, extrusion	Enzymatic	n/a	A: Rapid gelation, high cellular adhesiveness, and promotion of cell migration and proliferation D: Insufficient mechanical properties for structural support and fast degradation	[118,119]
	dECM	Extrusion	Thermal	>95%	A: Suitable biomimicry, promotion of cell differentiation, proliferation, and long-term functionality D: Slow gelation and weak mechanical properties	[93,105,120,121,122,123,124]
